# Magnetic character of holmium atom adsorbed on platinum surface

**DOI:** 10.1038/s41598-017-02809-7

**Published:** 2017-06-05

**Authors:** A. B. Shick, D. S. Shapiro, J. Kolorenc, A. I. Lichtenstein

**Affiliations:** 1 Institute of Physics, Czech Academy of Sciences, Na Slovance 2, CZ-18221 Prague, Czech Republic; 2 V. A. Kotel’nikov Institute of Radio Engineering and Electronics, Russian Academy of Sciences, Moscow, 125009 Russia; 3 Moscow Institute of Physics and Technology, Dolgoprudny, Moscow Region, 141700 Russia; 4 University of Hamburg, Jungiusstrasse 9, 20355 Hamburg, Germany

## Abstract

We address a recent controversy concerning the magnetic state of holmium adatom on platinum surface. Within a combination of the density functional theory (DFT) with the exact diagonalization (ED) of Anderson impurity model, the 〈*J*_*z*_〉 = 0 paramagnetic ground state |*J* = 8, *J*_*z*_ = ±8〉 is found. In an external magnetic field, this state is transformed to a spin-polarized state with 〈*J*_*z*_〉 ≈ 6.7. We emphasize the role of 5*d*–4*f* interorbital exchange polarization in modification of the 4*f* shell energy spectrum.

## Introduction

The study of single magnetic rare-earth (RE) atoms adsorbed on metallic^1–3^ and insulating^4^ solid surfaces recently became a subject of intense research. These “single-atom” magnets serve as benchmarks in a quest for the ultimate size limit of magnetic information storage. The major advance of observing the magnetic remanence was recently reported for the Ho adatom on MgO substrate^4^. The stable magnetic quantum state of the adatom was found on the time scale of 1500 s at 10 K temperature.

There is an ongoing debate whether the magnetic moment on Ho atom on Pt(111) surface (Ho@Pt) can be stable on the long time scale. Recent inelastic electron tunneling spectroscopy (IETS) measurements^1^ reported the moment lifetime up to 700 s below 1 K temperature, due to the single-ion magnetic anisotropy. However, the x-ray spectroscopy experiments^2^ have shown that the ground state of Ho is magnetically unstable, with no magnetic remanence. Moreover, the newer IETS experimental data^3^ did not see neither signatures of the spin-flip excitations nor spin-based telegraph noise for Ho atoms. This indicates that the 4*f* electrons do not contribute to the spin polarized tunneling processes in RE atoms on metals.

Theoretical calculations can shed light on the controversy concerning the magnetic state of Ho@Pt. The two |*J* = 8, *J*_*z*_ = ±8〉 magnetic ground states pointing into and out of the Pt(111) surface were inferred from *ab initio* density functional theory (DFT) calculations^1^. The multiplet calculations^2^ with the parameters chosen to reproduce the x-ray magnetic circular dichroism (XMCD) spectra resulted in |*J* = 8, *J*_*z*_ = ±6〉 ground states. Further analysis^5^ critically reexamined the XMCD data analysis^2^, and confirmed qualitatively the magnetically unstable ground state of Ho.

Up to date, conventional DFT and DFT+Coulomb *U* ^6, 7^ (DFT+U) methodologies were used in the calculations of Ho@Pt^1, 3^. Their main role was to identify the most favorable adsorption site and the optimal height for the Ho adatom above the Pt surface layer. While DFT+U can describe the chemical inertness of the 4*f* shell, it does not include the atomic multiplet effects, and can yield ambiguous results for the magnetic moments, or valence stability^8^. Recently, the combination of DFT with the dynamical mean field theory^9^ in a form of the Hubbard-I approximation (HIA)^10^ has been applied to the elemental RE, and is shown to be superior to the DFT+U and semiempirical ligand field (or equvalently crystal field) theory^11^. It opens new opportunities to treat the electronic structure of complex materials containing RE elements.

Here, we report the charge self-consistent electronic structure theory of Ho@Pt performed by combining DFT with the exact diagonalization (ED)^12^ of a single-impurity Anderson model^13^. In this approach, the DFT electronic structure obtained by the relativistic version^14, 15^ (with the spin-orbit coupling (SOC) included) of the full-potential linearized augmented plane wave method (FP-LAPW)^16^ is consistently extended to account for the full structure of the 4*f*-orbital atomic multiplets and their interaction with the conduction bands^17^. Previously, the method was used to treat the 4*f*-electron materials in paramagnetic phase^8, 18^, and we extend it to the spin-polarized case.

## Methodology

We use the 3 × 3 × 1 supercell model with twenty seven Pt atoms (three layers), and the rare-earth adatom which is placed either in the *hcp*(-Ho@Pt) or the *fcc*(-Ho@Pt) hollow positions atop the Pt(111) surface (see supplemental Fig. S1). The symmetry of both adsorption sites is *C*_3*v*_. The difference between *hcp*-Ho@Pt and *fcc*-Ho@Pt originates from the different placement of the Pt atoms in the sub-surface layer. The optimal heights for the rare-earth Ho adatoms above the Pt surface layer are taken from ref. 1 as *h*_*hcp*_ = 4.386 bohr and *h*_*fcc*_ = 4.348 bohr.

In the RE atoms the 4*f* electrons are mainly responsible for the magnetic moment, and the external *spd* electrons make only a discreet contribution to it. Their role, however, can not be disregarded, since these outer electrons strongly infuence the electronic and magnetic properties of the system. Therefore, we consider the multi-orbital Hamiltonian^10^ *H* = *H* ^0^ + *H* ^int^. *H* ^0^ is the one-particle Hamiltonian found from *ab initio* electronic structure calculations of a supercell; *H* ^int^ is the on-site Coulomb interaction^10^ describing the *f*-electron correlation. We assume that electron interactions in the *s*, *p*, and *d* shells are well approximated in DFT.

The DFT+ED calculations are performed in the charge self-consistent implementation described in section “Theoretical methods and computational details”. The effects of the interaction Hamiltonian *H* ^int^ on the electronic structure are accounted by a one-particle selfenergy Σ, which is constructed with the aid of an auxiliary impurity model Eq. (1) describing the complete seven-orbital 4*f* shell. The band Lanczos method^12^ is employed to find the lowest-lying eigenstates of the many-body Hamiltonian *H*_imp_ and to calculate the one-particle Green’s function *G*_imp_ and the selfenergy Σ in the subspace of the localized *f* orbitals {*ϕ*_*γ*_} at low temperature (*k*_B_ *T* = 1/500 eV). The Coulomb *U* values of 7.03 eV, and the exchange *J* of 0.83 eV were used, which are in the ballpark of commonly accepted values of *U* and *J* for the rare earths^19, 20^.

## Results

The XMCD measurements^2^ are performed in an external magnetic field (up to 7 T) assuming the magnetic saturation of the Ho@Pt system. Therefore, we have performed the spin-polarized calculations assuming the magnetic saturation of the Ho-adatom *f*-shell. In these calculations, we applied HIA and ED to DFT with the non-spin-polarized exchange-correlation functional^21^ in order to exclude the contribution of *f*-intraorbital exchange field into the double-counting *W*_dc_^22^. The exchange splitting Δ_ex_ in Eq. (1), which corresponds to the interorbital exchange energy between the 4*f* and 5*d* states of Ho, was varied as a parameter in a range from 5 meV to 15 meV (see the section “Theoretical methods and computational details” for further explanation of the Δ_ex_ choice). We have examined that it is enough to produce a fully spin-polarized solution and the selfenergy in Eq. (1).

First, we discuss the spin-polarized DFT+ED for the *fcc*-Ho@Pt. The *f*-states occupation 〈*n*_*f*_〉 changes only a little - from 10.17 to 10.16 - with the change of Δ_ex_. The calculated ground state magnetic properties are shown in Table 1 in comparison with the XMCD^2^ experiments. The values of spin moment ${M}_{S}=-2\langle {S}_{z}\rangle {\mu }_{B}/\hslash$ change only a little with the change of Δ_ex_. The changes in orbital moment ${M}_{L}=-\langle {L}_{z}\rangle {\mu }_{B}/\hslash$, and the magnetic dipole moment ${M}_{D}=-6\langle {T}_{z}\rangle {\mu }_{B}/\hslash$ are somewhat bigger indicating enhancement of the orbital polarization with an increase of Δ_ex_. Due to simultaneous increase of the orbital *M*_*L*_ and the the magnetic dipole *M*_*D*_ moments, the ratio ${R}_{LS}=\tfrac{{M}_{L}}{{M}_{S}+{M}_{D}}=1.20$ does not change with an increase of Δ_ex_. It is somewhat smaller than the XMCD result^2^ *R*_*LS*_ 1.51 ± 0.09, but still in reasonable agreement with the experiment. The magnitude of the total moment $\langle {J}_{z}\rangle ={M}_{S}\mathrm{/2}+{M}_{L}$ of 6.6 (Δ_ex_ = 5 meV), and 6.8 (10–15 meV) exceeds somewhat the experimental 〈*J*_*z*_〉 of 5.34–5.50^2^. It is closer to 〈*J*_*z*_〉 = 6^2^ obtained in the multiplet calculations than to 〈*J*_*z*_〉 = 8^1^ inferred from DFT+U. Note that in the presence of the crystal-field interaction, the values of the magnetic moment *M*_*J*_ = *M*_*S*_ + *M*_*L*_ obtained from the data of Table 1 are smaller than the maximum magnetic moment *g*_*J*_ *J* = 9.84, where *g*_*J*_ = 1.23 is the Lande factor and *J* = 8.00 is the expectation value of the total moment calculated for the ground state of Eq. (1). No noticeable differences are found for Ho@Pt in *fcc* and *hcp* positions (see supplemental Table S2).

**Table 1:** Spin (*M*_*S*_), orbital (*M*_*L*_), *M*_*S*_ plus magnetic dipole *M*_*D*_ moments (in *μ*_*B*_), and the total 〈*J*_*z*_〉 = *M*_*S*_/2 + *M*_*L*_ for the *fcc*-Ho adatom on Pt(111) with different values of the exchange splitting Δ_ex_, in comparison with DFT+U^1, 3^ and experimental data^2^.

	〈*M*_*S*_〉	〈*M*_*L*_〉	〈*M*_*S*_〉 + 〈*M*_*D*_〉	〈*J*_*z*_〉
DFT+U^1^	4.1	5.6	—	7.65
DFT+U^3^	3.91	5.88	—	7.84
Δ_ex_ = 5 meV	3.39	4.92	4.09	6.62
Δ_ex_ = 10 meV	3.32	5.14	4.28	6.80
Δ_ex_ = 15 meV	3.32	5.15	4.30	6.82
XMCD^2^	2.28 ± 0.12	4.28 ± 0.06	2.84 ± 0.13	5.42 ± 0.08

**Figure 1:**
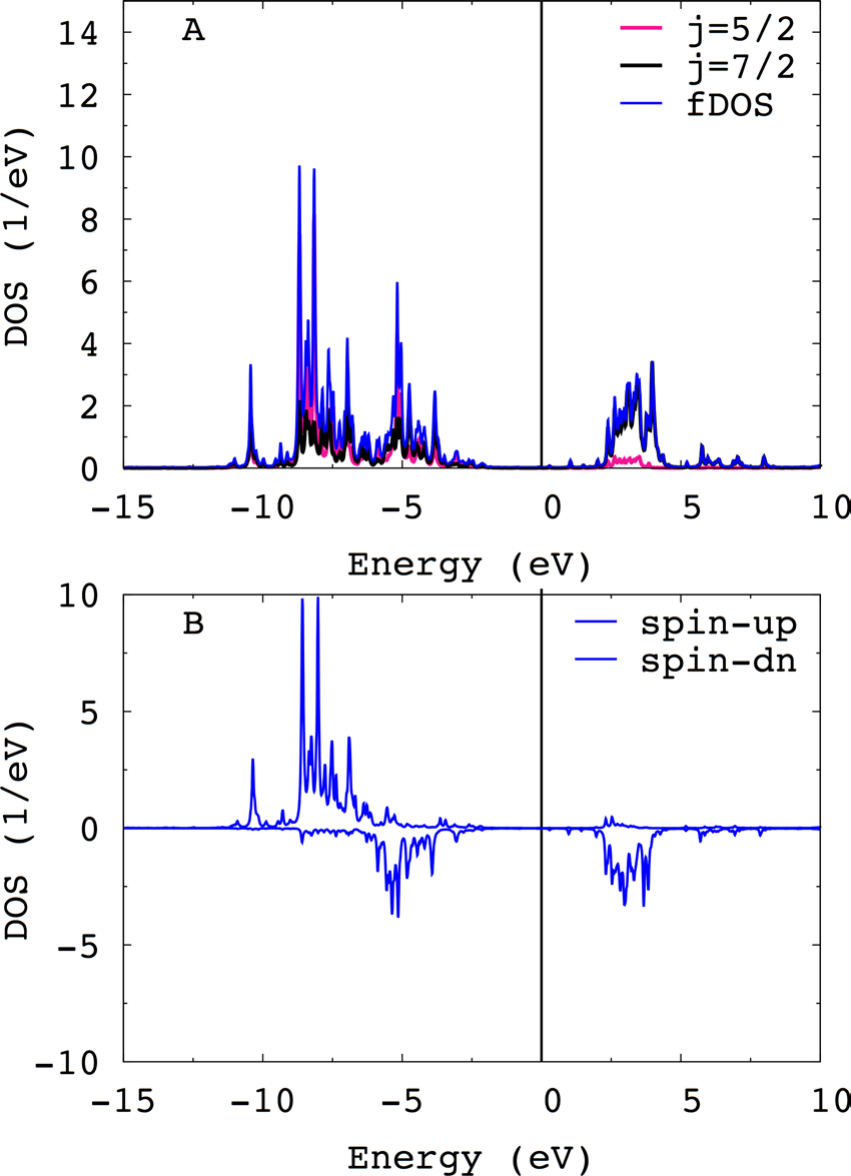
The total and *j* = 5/2, 7/2 projected fDOS for the fcc-Ho@Pt(111) (**A**); the spin projected fDOS for the fcc-Ho@Pt(111) (**B**).

The total and *j* = 5/2, 7/2 projected *f* orbital density of states (fDOS) for the spin-polarized *fcc*-Ho@Pt and Δ_ex_ = 10 meV is shown in Fig. 1(A). DFT+ED yields for the occupied 4*f*-states the first multiplet peak at ~4 eV below *E*_*F*_, and for the empty states at ~2.5 eV, consistent with the bulk hcp-Ho PES^23^. Effect of spin-polarization is illlustrated in Fig. 1(B) where the spin-resolved fDOS is shown. The spin-↑ intensities lie at ~7–8 eV below *E*_*F*_, and the spin-↓ at ~4–6 eV below *E*_*F*_. The empty states of spectrum at ~2–4 eV are practically fully spin-polarized. There is no surprise that these states are dominated by the *j* = 7/2 contribution since the *j* = 5/2 states are fully occupied for the *f* ^10^ manifold.

**Figure 2:**
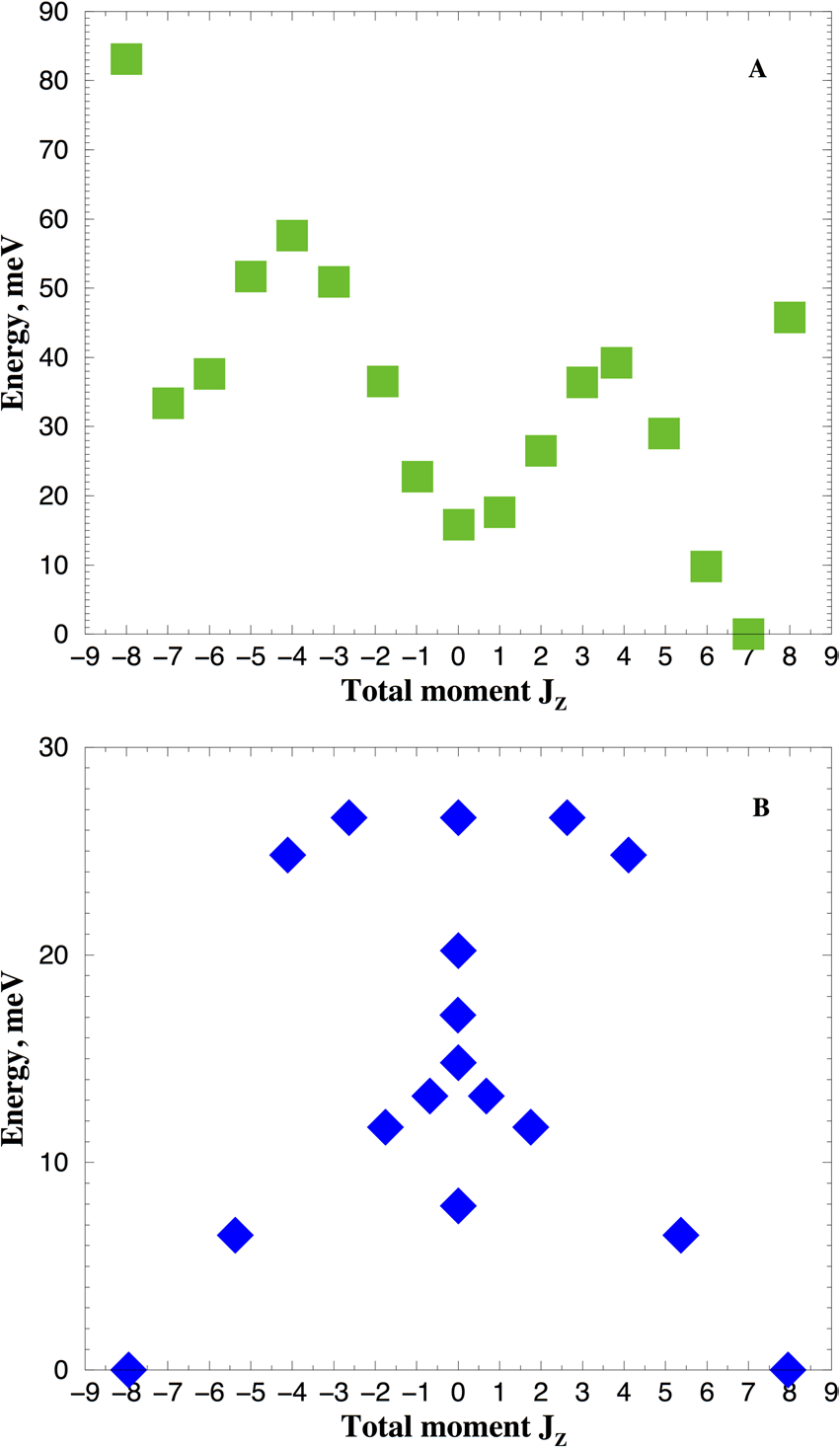
Scheme of quantum many-body levels of the lowest *J*_*f*_ = 8.00 multiplet obtained in Eq. (1) with the Δ_*CF*_ parameters for spin-polarized calculations and Δ_*ex*_ = 10 meV (**A**); with the Δ_*CF*_ parameters for non-spin-polarized calculations (**B**).

The energy splitting of the seventeen lowest many-body eigenvalues of Eq. (1), which correspond to the expectation value of *J*_*f*_ = 8.00, are shown in Fig. 2(A) (also see supplemental Table S3). The lowest energy is $|J=8,{J}_{z}=7.00\rangle$ state, next to it is the state $|J=8,{J}_{z}=5.98\rangle$ which is 10 meV higher in the energy, and the third state is $|J=8,{J}_{z}=0.02\rangle$, higher in the energy by 6 meV. This energy difference determines the magnetic anisotropy energy (MAE) barrier of 16 meV to turn the magnetization from the out-of-plane to the in-plane orientation. This value is about three times smaller than the MAE obtained by DFT+U^1^.

The experimental IETS results^1^ yield the spin excitations at the 5 meV and 8 meV energies. However, in the further experiments^3^, no signature of inelastic signal distinguishable from the substrate spectrum was found. We have calculated the model IETS spectra for the polarized *fcc*-Ho@Pt as given in the Supplemental material. We found shallow steps at the low energies ±10 meV, and other steps at the energies over 40 meV. Thus, our results disagree with earlier experiments of Miyamachi *et al*.^1^. Whether the calculated steps can be seen in the experiments depends on the intensity of the differential conductance (see the Eq. (S3) in Supplemental Material). Since it is proportional to the very small hybridization strength ~17 meV, the IETS intensity will be further reduced. We conclude that our results do not contradict qualitatively the experimental findings^3^.

Once the magnetic field is switched off, the adatom becomes non-magnetic. The self-consistent solution of Eq. (3) for paramagnetic state (Δ_ex_ = 0) represents the final state after the demagnetization. In this state, the *f*-electron count 〈*n*_*f*_〉 changes to 10.5, and the crystal field changes as well (see supplemental Table S4 for comparison between spin-polarized and paramagnetic Δ_CF_). It is not surprising, since the symmetry of the many-body ground state of Eq. (1) is changing, inducing the changes in the local occupation matrix ${n}_{{\gamma }_{1}{\gamma }_{2}}$ and corresponding effective LDA+*U* potential *V*_*U*_ in Eq. (3). This effect is neglected in semiempirical ligand field theory.

The energy splitting of the seventeen lowest many-body eigenvalues of Eq. (1) in paramagnetic state are shown in Fig. 2(C)) (also see supplementary Table S3). The lowest energy is the $|J=8,{J}_{z}=\pm 7.95\rangle$ state, in agreement with 〈*J*_*z*_〉 = ±8^1^. Next to it is the state $|J=8,{J}_{z}=\pm 5.36\rangle$ which is 6.5 meV higher in the energy, and the second exited state is $|J=8,{J}_{z}=0.0\rangle$, higher in the energy by 1.4 meV. This energy difference determines the so-called zero-field splitting energy barrier of 7.9 meV, which is twice as large as given by the ligand field theory^2^. Note, that application of the external magnetic field of 7 T to this paramagnetic state in Eq. (1) will not produce the spin-polarized solution shown in Fig. 2(A), since the parameters of the Eq. (1) hamiltonian are different (Δ_CF_, Δ_ex_, *e*_*f*_). This difference is determined by the charge self-consistency, and the 5*d*–4*f* interorbital exchange coupling. This spin-polarized ground state can not be described by simple re-population of the Zeeman-split non-magnetic many-body levels.

The transition from the initial magnetically polarized state to the final paramagnetic state in the quantum regime is a complex problem^24^ and we leave the theoretical description of the magnetization dynamics for future considerations. Nonetheless, we notice that if we assume Δ_ex_ = 0 while keeping all other parameters in Eq. (1) the same as in the spin-polarized case, the ground state changes to $|J=8,{J}_{z}=0\rangle$ and the adatom becomes magnetically unstable (see Supplemental Material).

## Conclusions

To summarize, comparison between spin-polarized and paramagnetic DFT+ED solutions for Ho@Pt shows that the correct 4*f* magnetic state in the presence of the external magnetic field can not be correctly described by simple re-population of the Zeeman-split many-body levels of a non-magnetic semiempirical hamiltonian. It is due to non-negligible role of the interaction between 4*f* and 5*d* electrons. We emphasize the role of 5*d*–4*f* interorbital exchange polarization in modification of the 4*f* shell energy spectrum, and overrule the existence of the magnetic 〈*J*_*z*_〉 = 8 ground state^1^.

## Theoretical Method and Computational Details

The multi-orbital impurity solver includes the full spherically symmetric Coulomb interaction, the spin-orbit coupling (SOC), the crystal field term (CF) describing the Coulomb interaction of the *f*-shell with other electrons, and the inter-orbital exchange field acting on the *f*-shell from other electrons. The corresponding Hamiltonian can be written as ref. 13

1 $$\begin{array}{rcl}{H}_{{\rm{imp}}} & = & \sum _{\begin{array}{c}kmm^{\prime} \\ \sigma \sigma ^{\prime} \end{array}}{[{\epsilon }^{k}]}_{mm^{\prime} }^{\sigma \,\sigma ^{\prime} }{b}_{km\sigma }^{\dagger }{b}_{km^{\prime} \sigma ^{\prime} }+\sum _{m\sigma }{\epsilon }_{f}{f}_{m\sigma }^{\dagger }{f}_{m\sigma }\\  &  & +\sum _{mm^{\prime} \sigma \sigma ^{\prime} }{[\xi {\bf{l}}\cdot {\bf{s}}+{{\rm{\Delta }}}_{{\rm{CF}}}+{{\rm{\Delta }}}_{{\rm{ex}}}{s}_{z}]}_{mm^{\prime} }^{\sigma \,\sigma ^{\prime} }{f}_{m\sigma }^{\dagger }{f}_{m^{\prime} \sigma ^{\prime} }\\  &  & +\sum _{\begin{array}{c}kmm^{\prime} \\ \sigma \sigma ^{\prime} \end{array}}({[{V}^{k}]}_{mm^{\prime} }^{\sigma \,\sigma ^{\prime} }{f}_{m\sigma }^{\dagger }{b}_{km^{\prime} \sigma ^{\prime} }+{\rm{h}}.{\rm{c}}.)\\  &  & +\frac{1}{2}\sum _{\begin{array}{c}mm^{\prime} m^{\prime\prime} \\ m^{\prime\prime\prime} \sigma \sigma ^{\prime} \end{array}}{U}_{mm^{\prime} m^{\prime\prime} m^{\prime\prime\prime} }{f}_{m\sigma }^{\dagger }{f}_{m^{\prime} \sigma ^{\prime} }^{\dagger }{f}_{m^{\prime\prime\prime} \sigma ^{\prime} }{f}_{m^{\prime\prime} \sigma ^{\prime} },\end{array}$$

where ${f}_{m\sigma }^{\dagger }$ creates an electron in the 4*f* shell and ${b}_{m\sigma }^{\dagger }$ creates an electron in the “bath” that consists of those host-band states that hybridize with the impurity 4*f* shell. The energy position ${\epsilon }_{f}$ of the impurity level, and the bath energies ${\epsilon }^{k}$ are measured from the chemical potential *μ*. The parameters *ξ*, Δ_ex_, and matrix Δ_CF_ specify the strength of the SOC, the exchange field, and the size of CF, acting on the *f*-shell. The parameter matrices *V* ^*k*^ describe the hybridization between the 4*f* states and the bath orbitals at energy ${\epsilon }^{k}$. The bath parameters *V* ^*k*^ and ${\epsilon }^{k}$ are determined from the LDA Green function *G*_LDA_(*z*) as described in ref. 18, and are shown in supplemental Table S1. The Ho *f*-shell SOC parameter *ξ* = 0.28 eV in Eq. (1) was determined from LDA calculations.

The exchange splitting Δ_ex_ in Eq. (1) corresponds to the interorbital exchange energy between the 4*f* and mainly 5*d* states of Ho, *J*_fd_ *m*_5*d*_^22^, where *J*_fd_ is ~0.1 eV^25^, and *m*_5*d*_ is the magnetic moment of the 5*d* states. As follows from the DFT and DFT+U calculations^3^ as well as from our own calculations, the *m*_5*d*_ does not exceed 0.1 *μ*_*B*_. It sets Δ_ex_ = 10 meV as an energy scale for the *J*_fd_ *m*_5*d*_. Note that Δ_ex_ exceeds the maximum external magnetic field value (0.4 meV) used in the XMCD experiments by an order of magnitude, and we did not include this field in the calculations.

The many-body Hamiltonian *H*_imp_ is solved employing the band Lanczos method^12^ and the selfenergy is obtained. The local Green’s function *G*(*z*) in the subspace of the localized spinorbitals {*ϕ*_*γ*_, *γ* = (*lmσ*)}, defining the *f* manifold of the rare-earth adatom, is calculated^17^ as

2 $$G(z)={[{G}_{0}^{-1}(z)+{\rm{\Delta }}{\epsilon }_{\sigma }-{\rm{\Sigma }}(z)]}^{-1},$$

where *G*_0_(*z*) is the non-interacting Green’s function, and ${\rm{\Delta }}{\epsilon }_{\sigma }$ are chosen to ensure the ${n}_{f}^{\sigma }$ occupations equal to the given number of spin-↑, ↓ correlated electrons. The matrix ${n}_{{\gamma }_{1}{\gamma }_{2}}=-\frac{1}{\pi }\,{\rm{Im}}{\int }_{-\infty }^{{E}_{{\rm{F}}}}\,{\rm{d}}z\,{[G(z)]}_{{\gamma }_{1}{\gamma }_{2}}$ is used to construct an effective LDA+*U* potential *V*_*U*_, which is inserted into Kohn–Sham-like equations:

3 $$[-{\nabla }^{2}+{V}_{{\rm{LDA}}}({\bf{r}})+{V}_{U}+\xi ({\bf{l}}\cdot {\bf{s}})]\,{{\rm{\Phi }}}_{{\bf{k}}}^{b}({\bf{r}})={\epsilon }_{{\bf{k}}}^{b}{{\rm{\Phi }}}_{{\bf{k}}}^{b}({\bf{r}}).$$

Note that the DFT contributions to the effective potential *V*_LDA_ in Eq. (3) are corrected to exclude the double-counting of the *f*-states non-spherical contributions to the DFT and DFT+U parts of the potential^17^.

These equations are iteratively solved until self-consistency over the charge density is reached. In each iteration, a new Green’s function *G*_0_(*z*), and a new value of the 4*f*-shell occupation are obtained from the solution of Eq. (3). Subsequently, a new selfenergy Σ(*z*) corresponding to the updated 4*f*-shell occupation is constructed. Finally, the next iteration is started by evaluating the new local Green’s function, Eq. (2). The self-consistent procedure defined by Eqs 1–3 was repeated until the convergence of the 4*f*-manifold occupations ${n}_{f}^{\uparrow ,\downarrow }$ was better than 0.01.

The CF matrix Δ_CF_ in Eq. (1) is obtained by projecting the solutions of Eq. (3) into the {*ϕ*_*γ*_} local *f*-shell basis,

4 $${[H]}_{{\gamma }_{1}{\gamma }_{2}}={\int }_{-\infty }^{+\infty }\,{\rm{d}}\epsilon \epsilon {[N(\epsilon )]}_{{\gamma }_{1}{\gamma }_{2}},$$

where, ${[N(\epsilon )]}_{{\gamma }_{1}{\gamma }_{2}}$ is an f-projected density of states (fDOS) matrix. The matrix elements of Δ_C*F*_ are then obtained by removing the interacting LDA+*U* potential ${[{V}_{U}]}_{{\gamma }_{1}{\gamma }_{2}}$ and SOC ${[\xi {\bf{l}}\cdot {\bf{s}}]}_{{\gamma }_{1}{\gamma }_{2}}$ from the local hamiltonian Eq. (4).

Since some electron-electron interaction energy is already included in LDA, the potential *V*_*U*_ in Eq. (3) includes the so-called double-counting correction *W*_dc_. Due to a self-consistency condition ${\epsilon }_{f}=-{W}_{dc}$ ^17^, it determines the mean position of the interacting *f*-level in Eq. (1), and controls the number of *f*-electrons. For the bulk Ho, the positions and the spectral shape of the occupied 4*f*-states are in a reasonable agreement with experimental valence-band photoelectron spectroscopy (PES)^23^, when the “around-mean-field” (AMF)^6^ flavour for *W*_dc_ is used (see Supplemental Material). Therefore, we used this *W*_dc_ in the Ho@Pt calculations.

## Electronic supplementary material

Supplementary Information
