# Supplementary material for: Magnetic character of holmium atom adsorbed on platinum surface
Source: Sci Rep. 2017 Jun 5;7:2751. doi: 10.1038/s41598-017-02809-7 (PMC5459836; doi:10.1038/s41598-017-02809-7)
Supplement: Supplementary file 1 — Supplementary Information [file 41598_2017_2809_MOESM1_ESM.pdf]

Supplemental Material for

Magnetic character of holmium atom adsorbed on platinum surface

A. B. Shick,[1] D. S. Shapiro,[2,3] J. Kolorenc,[1] and A. I. Lichtenstein[4]

[1] *Institute of Physics, Czech Academy of Sciences, Na Slovance 2, CZ-18221 Prague, Czech Republic*

[2] *Institute of Radio Engineering and Electronics, RAS, 125009 Moscow, Russia*

[3] *Moscow Institute of Physics and Technology, Dolgoprudny, Moscow Region 141700, Russia*

[4] *University of Hamburg, Jungiusstrasse 9, 20355 Hamburg, Germany*

SUPERCELL MODEL

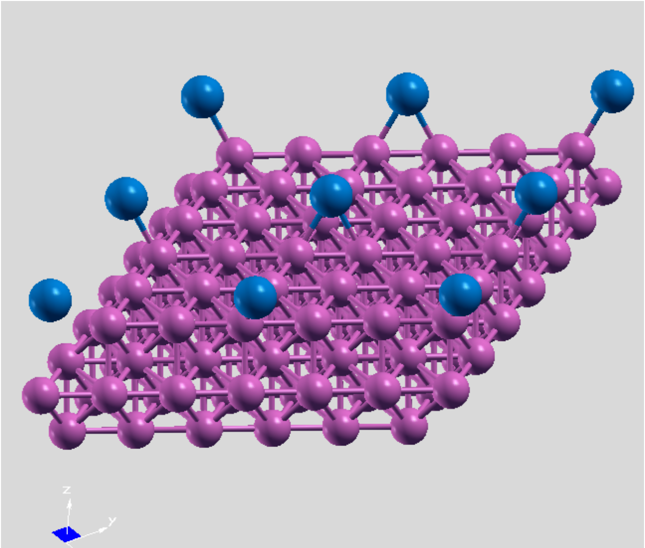

FIG. S1: (Color online) Schematic supercell model for Ho adatom on Pt(111).

BATH STATE PARAMETERS USED IN DFT+ED

TABLE S1: $4f$-states occupations $n_f^{5/2}$ and $n_f^{7/2}$, and bath state parameters $\epsilon^1_{5/2,7/2}$ (eV), $V^1_{5/2,7/2}$ (eV) for bulk Ho, and Ho adatom on Pt(111) surface in the *hcp* and *fcc* positions from LDA calculations.

Material	$n_f^{5/2}$	$n_f^{7/2}$	$\epsilon_1^{5/2}$	$V_1^{5/2}$	$\epsilon_1^{7/2}$	$V_1^{7/2}$
hcp-Ho-bulk	5.99	4.56	-0.09	0.15	0.105	0.11
hcp-Ho@Pt	5.93	4.62	0.20	0.095	-0.06	0.087
fcc-Ho@Pt	5.93	4.73	0.25	0.097	-0.065	0.087

CHOICE OF THE DOUBLE-COUNTING

No unique choice of the double counting exists in DFT+U and DFT+ED calculations, and “physical” arguments prevail in the choice of $W_{\rm dc}$. In previously reported LDA+Hubbard I calculations (HIA) for the bulk Ho [1], the $W_{\rm dc}$ was fixed to adjust the position of the first multiplet peak below E_F, which was measured by experimental valence-band photoelectron spectroscopy (PES) [2].

Since no PES results exist for Ho@Pt, we assumed the transferability of W_{dc}, and performed the DFT+ED calculations for the bulk Ho in the paramagnetic state (Δ_{ex}=0 in Eq. (1) of the main text) making use of the experimental lattice parameter value a and the c/a ratio . The resulting densities of state (DOS) obtained in DFT+ED with "fully localized" limit (FLL) [3] and "around-mean-field" (AMF) [4] flavours for W_{dc} are shown in Fig. S2. The FLL results are in striking disagreement with the experimental data [2]. DFT+ED-AMF gives the positions and the spectral shape of the occupied $4f$-states in a reasonable agreement with the experiment (first multiplet peak at 3.3 eV below E_F), and a bit worse for the empty $4f$-states (first multiplet peak at 3.8 eV above E_F). Comparison with experimental valence-band photoelectron spectroscopy (PES) is often taken as important criterion of truthfulness of electronic structure calculations. Thus, we conclude that AMF flavour is a better approximation, and places the $4f$-level ϵ_f in Eq. (1) correctly. Therefore, this form of W_{dc} is used in the further calculations.

In Fig. S2C we show the total DOS per Ho atom for comparison with the results of [5]. There is a good agreement between these two calculations. The difference in the energy positions of the f-peaks is caused by a different choice of W_{dc}. In [5], it was adjusted so that the position of the first multiplet peak below E_F matched the experimental PES.

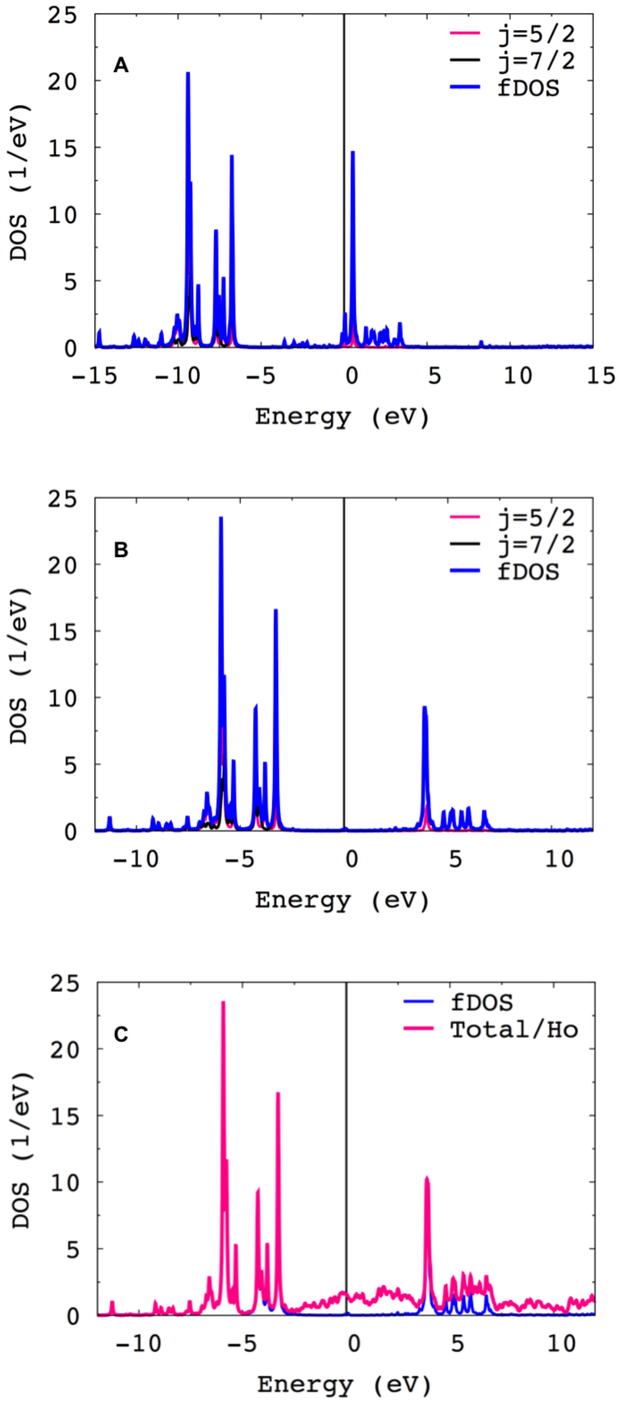

FIG. S2: (Color online) f-orbital DOS (total and $j = 5/2, 7/2$ projected) for the bulk hcp-Ho calculated with DFT+ED-FLL (A), DFT+ED-AMF (B), together with the total DOS/per Ho atom (C).

RESULTS OF DFT+HIA CALCULATIONS.

We performed the DFT+HIA calculations without hybridization term in Eq. (1) of the main text. For the fcc-Ho@Pt, the ground state of the Ho adatom, the solution of Eq. (3) of the main text, has $\langle n_f \rangle = 10.18$ f-electrons. The out-of-plane directed spin M_S, and the orbital M_L magnetic moments for the f-shell calculated using the

local Green function Eq. (2) are shown in Table S2, in comparison with previously reported DFT+U results of [6, 7]. Note that DFT+U results were obtained with the same Korringa-Kohn-Rostoker (KKR) method [8] and differ for a reason which is not completely clear. It is seen that the DFT+U results exceed substantially the experimental values for both M_S and M_L obtained in the XMCD experiments [9] shown in Table I of the main text. The use of DFT+HIA brings the results closer to the XMCD [9] data for both the spin M_S and the orbital M_L components of the total moment M_J. No noticeable differences are found for Ho@Pt in fcc and hcp positions.

TABLE S2: Spin (M_S), and orbital (M_L) magnetic moments (in μ_B) and $4f$ occupation n_f of the Ho adatom on Pt(111) from DFT+HIA calculations with Δ_{ex} = 10 meV.

**Site**	fcc-Ho@Pt			hcp-Ho@Pt		
	n_f	M_S	M_L	n_f	M_S	M_L
DFT+U [6]	N/A	4.1	5.6	N/A	3.9	5.45
DFT+U [7]	10.14	3.91	5.88	10.14	3.91	5.88
DFT+HIA	10.18	3.28	5.16	10.17	3.25	5.17

DETAILS OF THE DFT+ED CALCULATIONS.

Energy (meV)	$\langle L_z \rangle$	$\langle S_z \rangle$	$\langle J_z \rangle$
0.0	5.32	1.67	7.00
9.8	4.59	1.39	5.98
15.8	-0.01	0.02	0.02
17.6	0.72	0.28	1.00
22.8	-0.74	-0.24	-0.98
26.5	1.47	0.52	2.00
29.0	3.83	1.14	4.97
33.4	-5.33	-1.66	-6.99
36.4	2.25	0.74	2.99
36.5	-1.38	-0.44	-1.82
37.6	-4.62	-1.38	-6.00
39.2	2.92	0.89	3.82
45.8	6.13	1.86	8.00
50.9	-2.29	-0.70	-2.99
51.7	-3.88	-1.12	-5.00
57.6	-3.09	-0.90	-4.00
83.1	-6.13	-1.86	-8.00

Energy (meV)	$\langle L_z \rangle$	$\langle S_z \rangle$	$\langle J_z \rangle$
0.0	-6.08	-1.87	-7.95
0.0	6.08	1.87	7.95
6.5	-4.10	-1.26	-5.37
6.5	4.10	1.26	5.37
7.9	0.00	0.00	0.00
11.7	-1.34	-0.41	-1.75
11.7	1.34	0.41	1.75
13.2	-0.51	-0.17	-0.68
13.2	0.51	0.17	0.68
14.8	0.00	0.00	0.00
17.1	-0.01	0.00	-0.01
20.2	0.01	0.00	0.01
24.8	-3.16	-0.95	-4.11
24.8	3.16	0.95	4.11
26.6	0.00	0.00	0.00
26.6	-2.02	-0.61	-2.63
26.6	2.02	0.61	2.63

TABLE S3: (Color online) The magnetic moments and energies from Eq. (1) of the main text for spin-polarized case (left panel, also shown in Fig. 2(A) of the main text); for paramagnetic case (right panel, also shown in Fig. 2(B) of the main text).

$$\begin{bmatrix} 0.035 & 0.000 & 0.000 & 0.000 & 0.000 & 0.000 & 0.001 \\ 0.000 & 0.012 & 0.000 & 0.000 & 0.000 & 0.000 & 0.000 \\ 0.000 & 0.000 & 0.014 & 0.000 & 0.000 & 0.000 & 0.000 \\ 0.000 & 0.000 & 0.000 & -0.059 & 0.000 & 0.000 & 0.000 \\ 0.000 & 0.000 & 0.000 & 0.000 & 0.014 & 0.000 & 0.000 \\ 0.000 & 0.000 & 0.000 & 0.000 & 0.000 & 0.012 & 0.000 \\ 0.001 & 0.000 & 0.000 & 0.000 & 0.000 & 0.000 & 0.035 \end{bmatrix} \qquad \begin{bmatrix} -0.001 & 0.000 & 0.000 & 0.000 & 0.000 & 0.000 & -0.012 \\ 0.000 & -0.013 & 0.000 & 0.000 & 0.000 & 0.000 & 0.000 \\ 0.000 & 0.000 & 0.037 & 0.000 & 0.000 & 0.000 & 0.000 \\ 0.000 & 0.000 & 0.000 & -0.049 & 0.000 & 0.000 & 0.000 \\ 0.000 & 0.000 & 0.000 & 0.000 & 0.037 & 0.000 & 0.000 \\ 0.000 & 0.000 & 0.000 & 0.000 & 0.000 & -0.013 & 0.000 \\ -0.012 & 0.000 & 0.000 & 0.000 & 0.000 & 0.000 & -0.001 \end{bmatrix}$$

TABLE S4: (Color online) fcc-Ho@Pt(111): Self-consistent Δ_{CF} (eV) determined from the local hamiltonian Eq. (4) of the main text from the spin-polarized calculations with Δ_{ex}= 10 meV (left panel); from the paramagnetic calculations (right panel).

INELASTIC SCANNING TUNNELING SPECTRA

The IETS is described with the total hamiltonian of the form [10]

$$H = H_{tip} + H_{sur} + H_{imp} \ , \tag{S1}$$

where $H_{tip} = \sum_{k,\sigma} \epsilon^{(tip)}_{k,\sigma} a^{\dagger}_{k\sigma} a_{k\sigma}$ corresponds to the electrons in the tip with energies $\epsilon^{(tip)}_{k,\sigma}$ and $H_{sur} = \sum_{k,\sigma} \epsilon^{(sur)}_{k,\sigma} b^{\dagger}_{k\sigma} b_{k\sigma}$ describes the electrons in the surface with energies $\epsilon^{(sur)}_{k,\sigma}$. The adatom hamiltonian H_{imp} is given by Eq. (1) of the main text with eigenvalues ϵ_M and eigenvectors $|M\rangle$.

For the spin-flip tunneling,

$$H_{tun} = \sum_{\substack{\sigma\neq\sigma' \\ kk'}} \left((g-1)t_1 \vec{\tau}_{\sigma\sigma'} \vec{J}\right) \left(a^{\dagger}_{k\sigma} b_{k'\sigma'} + b^{\dagger}_{k'\sigma'} a_{k\sigma}\right) . \tag{S2}$$

In the above equation τ_α are the Pauli matrices, J_α are the total moment operators ($\alpha = x,\ y,\ z$), and g is the Lande factor. The t_1 tunnelling matrix element is proportional to the square of hybridization parameters V^k shown in Table S1.

The differential conductance $G = \frac{\partial I}{\partial V}$ is, [10]

$$G = G_S \sum_{\substack{MM' \\ s=\pm1}} P_M |\langle M|\vec{J}|M'\rangle|^2 \left(\frac{1}{1-e^{\beta(\Delta_{M'M}-sV)}} + \frac{\beta\,(\Delta_{M'M}-sV)}{4\sinh^2\frac{\beta(\Delta_{M'M}-sV)}{2}}\right) , \tag{S3}$$

where, $\Delta_{M'M} = \epsilon_{M'} - \epsilon_M$, P_M is the statistical weight of the eigenstate $|M\rangle$, and $G_S = (g-1)^2 t_1^2 \rho_{sur}(\epsilon_F)\rho_{tip}(\epsilon_F)$ proportional to the hybridization, and to the surface/tip $\rho_{sur/tip}$ DOS at the Fermi edge. V is an external voltage.

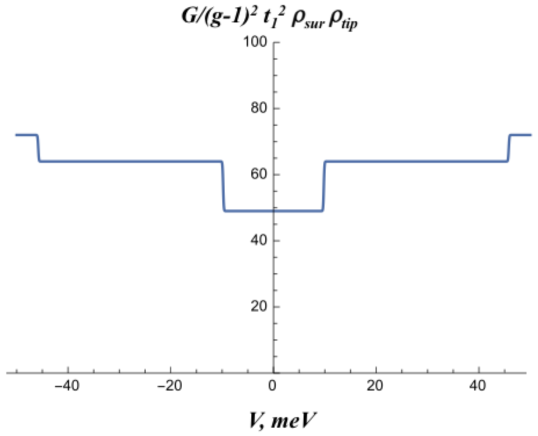

FIG. S3: (Color online) Spin excitation spectrum of Ho adatom on Pt(111).

The IETS spectrum for Ho adatom on Pt(111) in spin-polarized state (see Table S3) calculated using the model Eq. (S3) is shown in Fig. S3. It is seen that the calculation gives a shallow step at the voltage ±10 meV. Other step(s) occur at the higher energy range (over 40 meV).

The G_S in Eq. (S3) is proportional to the square of hybridization strength t_1 multiplied by a prefactor $(g-1)^2$ where g is the Lande factor. Assuming that the most important contribution to the tunnelling comes from the hybridization occurring in the vicinity of E_F, and taking into account that the $t_1 \sim |V_k|^2$ shown in Table S1, we obtain the hybridization strength in the range of 12-23 meV. The prefactor G_S is reduced further by $(g-1)^2 = 0.05$ where the Lande factor g=1.23 for $J = 8$ many body state.

SCENARIO FOR MAGNETIC INSTABILITY OF HO@PT(111)

After the magnetic field is removed, the magnetization starts to evolve. We keep in mind that the $4f$ shell and $5d$ states can react differently, and that the latter is demagnetized faster than the former. This assumption is qualitatively consistent with recent experimental observation of a difference, by one order of magnitude, in the ultrafast magnetic moment dynamics of the localized $4f$ and itinerant $5d$ states in the RE metals [11].

We model this magnetic moment evolution in a simplified picture: we assume $\Delta_{\text{ex}} = 0$ while keeping all other parameters in Eq. (1) of the main text the same as in the spin-polarized case. The energy splitting of the seventeen lowest many-body eigenvalues of Eq. (1) for this intermediate state are shown in Fig. S4. Importantly, the lowest eigenstate, $|J = 8, J_z = 0\rangle$, has zero moment and is almost degenerate (within 1 meV energy difference) with a doublet $|J = 8, J_z = \pm 6.98\rangle$. The zero-field splitting becomes negligibly small, meaning that the system can go in and out of the magnetic state at no energy cost, thus it becomes magnetically unstable. Since the transition to this intermediate state is connected with the "fast" demagnetization of the $5d$ shell, the Ho adatom becomes magnetically unstable before the f-shell "slowly" transforms to the final fully relaxed paramagnetic state.

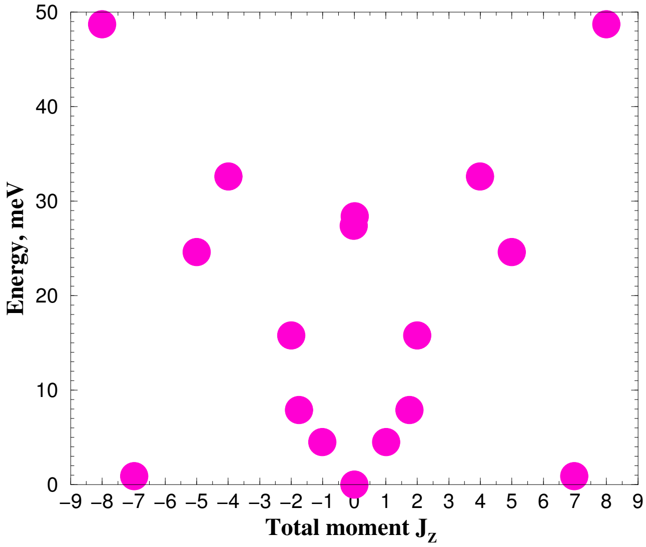

FIG. S4: (Color online) Scheme of quantum many-body levels of the lowest $J_f = 8.00$ multiplet obtained in Eq. (1) of the main text with the Δ_{CF} parameters for spin-polarized calculations and $\Delta_{ex} = 0$.

[1] S. Lebegue *et al.*, Phys. Rev. B **74**, 045114 (2006).
[2] S. Lang, Y. Baer, and P. Cox, J. Phys. F: Met. Phys. **11**, 121 (1981).
[3] A. Lichtenstein, V. I. Anisimov, and Zaanen, Phys. Rev. B **52**, R5467(R) (1995).
[4] V. I. Anisimov, J. Zaanen, and O. K. Andersen, Phys. Rev. B **44**, 943 (1991).
[5] I. L. M. Locht *et al*, Phys. Rev. B **94**, 085137 (2016).
[6] J. H. Miyamachi *et al.*, Nature **503**, 242 (2013).
[7] M. Steinbrecher *et al.*, Nature Communications **7**, 10454 (2016).
[8] R. Zeller and P. H. Dederichs, Phys. Rev. Lett. **42**, 1713 (1979).
[9] F. Donati *et al.*, Phys. Rev. Lett. **113**, 237201 (2014).
[10] J. Fernndez-Rossier, Phys Rev. Lett. **102**, 256802 (2009).
[11] B. Frietsch *et al.*,, Nature Commun. **6**, 8262 (2015).
